# The vampire in late communist cinema: from internal enemy to foreign threat

**DOI:** 10.1007/s11212-025-09734-w

**Published:** 2025-06-11

**Authors:** Anca Simina Martin

**Affiliations:** Lucian Blaga University of Sibiu, Sibiu, Romania

**Keywords:** Communism, Dracula, Soviet Union, Romania, Vampire

## Abstract

The vampire trope’s connection to socialism originates in Karl Marx’s *The Communist Manifesto* (1848), where it symbolizes capitalism. This interpretation has dominated vampire studies, often narrowing the discourse on the semantic dimensions of the vampire in communist states. This article examines the evolution of the vampire myth in Eastern bloc cinema from Lenin’s 1917 October Revolution to Romania’s 1989 communist collapse. By comparing socialist vampire movies to Western counterparts, the study reveals that Eastern European vampires usually embody the haunting legacy of communism’s earlier phases. The article’s final section contrasts vampire depictions in Poland, Czechoslovakia, Russia, and Romania during the cinema of late communism. While Romania—known as the vampire trope’s birthplace—avoided the myth in cinema, other countries embraced it during this period to critique consumerism and shifting gender roles in Soviet societies. Through this exploration, the article illuminates the cinematic interpretations of the vampire across different socialist contexts.

Discussions about the vampire trope in leftist discourse begin—and most often end (Gordon Melton 2011, pp. 673–4)—with the metaphor of the ‘undead’ material capital feeding on working-class ‘living’ labor in Karl Marx’s critique, particularly in the first volume of *Capital* (1867): “Capital is dead labour which, vampire-like, lives only by sucking living labour, and lives the more, the more labour it sucks” (1976, p. 342). Scholars’ insistence on commenting on this occurrence of the metaphor is easily understood; as Mark Neocleous aptly notes, “the vampire motif, if not the vampire himself, runs like a red thread through [Marx’s] work” (2003, pp. 670–1) appearing in writings predating *Capital*, such as *The Class Struggles in France, 1848 to 1850* (1850), and in notes published posthumously, such as the *Economic and Philosophic Manuscripts of 1844*, released in 1932 by the Soviet Union’s Marx–Engels–Lenin Institute. However, Marx’ concept of the vampire is not tied to any particular individual or group. Instead, it serves as a symbol of the broader capitalist system and its agents, whose insatiable drive for accumulation, in his view, echoed the monstrous thirst of the vampires depicted by Ernst T.A. Hoffmann and Alexandre Dumas père (Frayling 1992, p. 83).^1^ In January 1918, less than a year after the beginning of the October Revolution, the vampire was given a clear identity: for Vladimir Lenin, the foundational figure of Soviet Russia, Marx’s vampires are the *kulaki*, the landowning peasants, who “have been gathering the landed estates into their hands” and “continue to enslave the poor peasants” (1965, p. 58). However, by the end of 1917, disillusioned with Lenin’s revolution, Russian novelist and literary critic Dmitry Merezhkovsky wrote that “[a] vampire came out of the murdered Romanov autocracy—the Leninist autocracy” (Volkov 2015, p. 131).^2^ In other words, Marx’s metaphor of the exploitative vampire–human was dynamic from the outset, instrumentalized by both leaders and critics of the regimes that drew on Marxist ideology.

Thus, limiting the vampire trope in communism to its Marxist understanding is reductive, not least because the German thinker died before the release of Bram Stoker’s *Dracula* (1897), arguably the most influential work of vampire literature, which spawned a translation into Russian as early as 1902 (Berni 2021, p. 52) and an unconfirmed movie in 1920—the first cinema adaptation of the novel (Gordon Melton 2011, p. 282)—after Lenin’s October Revolution. This essay seeks to complement recent ideological analyses of horror in late communism, such as *Fear Before the Fall* (2023), by examining how vampire cinema specifically reflects the Eastern Bloc’s anxieties about the past—whether the specter of the Tsarist ruling class or oppressive leaders in the history of communism. In the 1980s, as the Iron Curtain began to crumble and populations in the communist countries increasingly looked toward the West—while the West itself became more aware of the Soviet Union’s failures—the vampire metaphor further evolved to reflect the shifting landscape of late communism, embodying the Eastern bloc’s struggle to navigate the uncertainty that lay beyond a fading half-century of authoritarian rule. Although one of the least prolific genres in communism (Nikulin 2019), vampire movies offer a unique perspective on the Eastern bloc’s anxieties during the 1980s, much as Western vampire fiction mirrored the American fear of “a designated enemy in the seemingly almighty Soviet Union” (Auerbach 1995, p. 6). However, to understand the peculiarities of vampire cinema in the wake of communism, it is important to explore the evolution of the myth against earlier phases in the history of socialism and the iterations of the trope in Western cinema.

## A brief history of vampire cinema in the eastern bloc: the vampiric aristocrat, exploiter and specter of past communist leaders

When the revolution finally broke out, numerous veteran Russian actors and directors escaped to places such as Odessa or Paris, fearing their position as part of the cultural elite (Herbert 2023, p. 13). Among them, however, were figures who would make history in Soviet Russia as well, such as Yakov Protazanov, the director of *Tanets s vampirom* (*Dance of the Vampire*) (1914) and *Satana likuyushchiy* (*Satan Triumphant*) (1917), the story of a priest falling prey to the devil’s temptation, which was released within weeks of the October Revolution. This movie, which “exemplified the marked turn toward apocalypticism in revolutionary Russian society,” is a prime example of the “decadent” cinema that the Bolsheviks criticized (Youngblood 2013, p. 25). However, sensationalist features continued to be made, albeit with horror elements less pronounced than in pre-Revolutionary productions such as *Satana likuyushchiy*. A notable example from this transitional period is the 1922 film *Prizrak brodit po Evrope* (*A Specter Haunts Europe*), which, though loosely adapted from Edgar Allan Poe’s 1842 short story “The Masque of the Red Death,” bears little resemblance to contemporary European horror films of the time, such as Friedrich W. Murnau’s *Nosferatu* (1922). Unlike *Nosferatu*, which draws inspiration from Bram Stoker’s *Dracula* and features the grotesque Count Orlok, *Prizrak brodit po Evrope* incorporates, in fact, themes from Marx’s *Communist Manifesto*, portraying an authoritarian emperor tormented by revolutionaries. *Medvezhya svadba* (*The Bear’s Wedding*), released in 1925 and based indirectly on a Western work,^3^ is less overt in its lip service to Marxist discourse but more vivid in its Gothic imagery, depicting the aristocratic antagonist as a bloodthirsty bear. What made possible its release was, on the one hand, the producers’ “pedigree”—its director Vladimir Gardin founded the Moscow Film School in 1919, and one of its authors was Anatoli Lunacharsky, who headed the State Commission on Education (Herbert 2023, p. 13)—and on the other, the fact that it concurred with Marx’s reformulation of the Gothic, revealing the pre-Revolution “aestheticized bodies of aristocrats” as bearing “signs of degeneration” (Bulgakowa 2019, p. 264).

Conversely, from the late 1920s to the early 1950s, which coincided with Stalin’s leadership, the government tightened its grip on the movie industry and the Party aimed to establish a new cinematic approach centered on realism (Herbert 2023, p. 15). Meanwhile, during the most challenging year of the Great Depression (Skal 1993, p. 115), the United States saw the release of Tod Browning’s *Dracula* (1931), starring Bela Lugosi in his iconic role as Count Dracula, “a sanguinary capitalist [who] relocates from Transylvania after draining the local peasants” (p. 159). In the Soviet Union, however, the film was criticized for endorsing the bourgeois and imperialist ideals of the West (Herbert 2023, p. 15). At first glance, this may seem nonsensical—after all, Lugosi’s villain is a “degenerate” aristocrat—but unlike the bloodsucker in *Medvezhya svadba*, he is also seductive, arousing fear and desire alike. Such portrayals, Russian critics argued, were “overly fixated on personal psychology and ignorant of class and social issues” (Herbert 2023, p. 14). The monstruous in early Soviet cinema did exist in the West—the 1940s saw the birth of the Lon Chaney Jr.’s *Wolf Man* (1941), a leading figure in Hollywood’s pantheon of horror monsters—but the Western iterations of the trope do not portray the working-class as the “New Man” socialists had in mind; instead, the werewolf is a “devolved animal-[man], often possessed of the wolfish traits so prized by the Nazis” (Skal 1993, p. 216), while “the Frankenstein monster is a poignant symbol for an army of abject and abandoned laborers” (p. 159).

The 1950s ushered in a wave of new anxieties, with one of the most widespread being the fear of nuclear destruction as Cold War tensions escalated worldwide (Jones 2023, p. 626). Moreover, the Soviet space program was shrouded in secrecy, “obscured by a figurative ‘space curtain’ that took much effort to see through” (Phelan 2012, p. 1). As a result, the Western sci-fi movies of the era emphasized the communists’ malevolence as a symptom of their “alien” nature (Hendershot 2001, p. 247). This aloofness makes young Marge Farrell realize that an unaffectionate extraterrestrial has taken her new husband’s shape in *I Married a Monster from Outer Space* (1958), a science-fiction reworking of an earlier feature, Robert Stevenson’s *I Married a Communist* (1949). In Soviet fiction, aliens are part of a socialist utopia, in which the humanoid Martians imagined by Alexander Bogdanov—the Bolshevik revolutionary and blood transfusion pioneer who wrote sci-fi novels—“exchange […] fluids, in free love and mutual blood transfusions” (Greenfield 2006, pp. 632–3). However, for this collective self-preservation to emerge—we learn from Bogdanov’s novel *Engineer Menni* (1913, translated into English in 1984)—journalist Teo, the defender of democracy and the Prime Minister, the protector of social peace on Mars had to be vampirized by Netti, the working-class vampire killer (Champine 2023, pp. 186–7); “[t]he blood of the old would fortify the blood of the young” (Greenfield 2006, p. 626) with “the accumulated knowledge of past eras,” “a vast wealth of raw materials to be reworked, reorganized into new systems of knowledge that are adapted to […] a changing environment” (Champine 2023, p. 187).

*I Married a Monster from Outer Space* is the alternate ending to *Engineer Menni*. In the latter, the Martian working-class understand that, in the future, the sun will die, and “joyfully welcome this moment, when the greatness of death will fuse with the greatest act of creation, the moment which will conclude our life only to pass its soul on to our brothers, whoever they may be” (Bogdanov 1984, p. 225). The Western movie, however, envisions the aliens from Mars—who possess vampire-like features such as living longer, having night vision and being impervious to bullets—as desperate, hoping to save their species by impregnating mortals after a solar radiation wiped out their women. In other words, this scenario materializes the most criticized strategy for survival in Bogdanov’s *Red Star* (1908, translated into English in 1984), his sequel to *Engineer Menni*, a vampiric invasion of planet Earth:

We must understand this necessity and look it squarely in the eye, however grim it might seem. We have only two alternatives: either we halt the development of our civilization, or we destroy the alien civilization on Earth. There is no third possibility. […] We must choose, and I say that we have but one choice. A higher form of life cannot be sacrificed for the sake of a lower one. Among all the people on Earth there are not even a few million who are consciously striving for a truly human type of life. For the sake of these embryonic human beings we cannot deny the birth and development of tens, maybe hundreds of millions of our own people, who are humans in an incomparably fuller sense of the word. We will not be guilty of cruelty, because we can destroy them with far less suffering than they are constantly causing each other. There is but one Life in the Universe, and it will be enriched rather than impoverished if it is our socialism rather than the distant, semi-barbaric Earthly variant that is allowed to develop, for thanks to its unbroken evolution and boundless potential, our life is infinitely more harmonious. (Bogdanov 1984, pp. 113, 116)

This proposal suggests that a socialist society cannot survive without dismantling the past, which is achieved either by “halting the development of our civilization” or self-destruction—the route taken by engineer Menni when faced with the risk of becoming a vampire—or by “destroy[ing] the alien civilization,” the forced removal strategy promoted by journalist Teo and the Prime Minister. However, this is not to say that the Bogdanov’s Martian socialist society has “come to a standstill” (Bogdanov 1984, p. 217). As Menni realizes in his conversation with the capitalist Vampire, to build a future aligned with the socialist cause means inflicting, with “[e]ach step, […] a blow to the past” (Bogdanov 1984, p. 225).

However, it should be noted that the utopian socialism Bogdanov envisioned is not about battling foreign forces, as seen in the case of the Martian who supports the vampiric invasion of Earth or in how American sci-fi horror movies portray the Cold War with Soviet Russia. Instead, it represents an internal struggle among native social classes. In this regard, Bogdanov’s novels about Mars, and *Engineer Menni* in particular, foreshadow Nikita Khrushchev’s de-Stalinization plan, which sought to exorcise Stalin’s cult of personality from the USSR. However, this process “was neither thorough nor genuinely cathartic” (Ryan 2009, p. 3); Stalin, “a living corpse, [was] preying vampire-like upon post-Stalinist […] society” (Ziolkowski 1998, p. 172).

In the late 1960s and 1970s, the growth of consumerism shifted the focus toward commercial interests (Herbert 2023, p. 16), while “the crushing of the Prague Spring [in 1968] accentuated the shift to a divisive ‘polycentrism’ in world communism” (McDermott and Stibbe 2018, p. 13). That the transition debuted earlier is testified, on the one hand, by the cinema readaptations of the horror literary works that were brought to screen in the early socialist period—Roman Tikhomirov’s 1960 *Pikovaya dama* (*The Queen of Spades*), Janusz Majewski’s 1970 *Lokis* in Poland, and Konstantin Yershov and Georgi Kropachyov’s *Viy* (1967), based on an 1835 novella with the same name by Nikolai Gogol. Though falsely credited as the only Soviet horror movie (McCorkle 2023, p. 722), *Viy*, the story of a young priest tormented by a witch, is nonetheless a steppingstone in the history of cinema, no less because it testifies to the fact that Soviet directors found it easier to circumvent censorship and explore the genre when the source material was folklore (Herbert 2023, p. 28). 4 This imagery, however, also served to allude reproachfully to the errors of past communist leaders; the vampires summoned by the sorceress “could be interpreted as a repressed memory of Stalinist faceless minions” (Dolgopolov 2014, p. 46). A similar critique exists in other features of the period, which appeared in less politically permissive contexts; Jaromil Jireš’s 1970 *Valerie a týden divů* (*Valerie and Her Week of Wonders*), released in the turbulent aftermath of the Prague Spring, tells the story of a thirteen-year-old peasant girl who is haunted by vampires, “an older generation that is irredeemably compromised by its traffic with dark forces—hypocritical, mendacious and corrupt” (Melville 2007). Ðorđe Kadijević’s *Leptirica* (*The She-Butterfly*), inspired from Milovan Glišić’s 1880 *Posle Devedeset Godina* (*After Ninety Years*), premiered in 1973 under similar circumstances, which saw the end of a relatively liberal period in the history of Yugoslav cinema. Like Jireš, Kadijević turned to folklore, with the peasant girl Radojka passing on the butterfly spirit of the vampire Sava Savanović to her husband-to-be; the young are doomed, *Leptirica* suggests, to repeat the oppressive ways of the past.

Despite the political context of their release, *Valerie a týden divů* and *Leptirica* are not fundamentally different from the Hammer movies, on which the British working-class and American students gorged during the 1960s and early 1970s (Auerbach 1995, p. 96). The former, for instance, “evokes the lusty peasant world of bosoms barely contained by ruffled white cotton that serves as counterpoint to undeath in so many Hammer productions” (Selavy 2008), while *Leptirica*’s mill-dwelling vampire is reminiscent of Baron Meinster in *The Brides of Dracula* (1960), who took refuge in a similar location, and Radojka falls prey to the vampire because the old woman guarding her failed, like the Baron’s mother, to prevent the vampiric abuse from happening. In other respects, the two vampire movies could not be more different from the features released around the same time in the West; when the vampire does not come from Transylvania, he hails from Serbia or Bulgaria,^5^ and his victims are primarily Westerners.^6^ However, some of these productions, while largely indebted to Stoker’s formula—the vampires are Eastern European aristocrats and their victims come from the West—deviate from it one very important respect, their open ending. That in Hammer Productions’ *Count Yorga, Vampire*, the movie ends with Los Angeles woman-turned-bloodsucker Donna attacking her boyfriend, Michael, and in *Il cav. Costante Nicosia*, the Italian heir’s baby son is revealed to have fangs “may be […] linked with [the] more ‘real’ horrors” lying beyond the Iron Curtain (Prawer 1990, p. 61).

“The combination of bloodsucking (to cover basic oppression) and work in the night (paralleling the secrecy of the Soviet security apparatus) makes the vampire an irresistible emblem of communist states,” writes Thomas Prasch in relation to *The Fearless Vampire Killers* (1967), which was released by Polish director Roman Polanski after taking refuge in the US from the Warsaw Pact (2016, p. 14). As seen above, this metaphor was also exploited on the other side of the Iron Curtain, with the amendment that, in the communist bloc, the vampire is always an internal rather than external threat, and almost always a stand-in for previous stages in the history of communist regime: if the vampire of Leninist and Stalinist Russia is the specter of the Tsarist rule, the vampire of the Space Race era—reanimated from early socialist works and juxtaposed onto the period’s Western vampire cinema—speaks of anxieties regarding the past, and the vampire of the post-Stalinist period embodies the long shadow cast by Stalin’s figure over the Eastern bloc. In the 1980s, almost thirty years following Stalin’s death, the trope would metamorphosize yet again to embody the communist states’ inability to reconcile their ideology with Western cultural trends.

## Vampiric consumerism and feminism in late communist vampire movies

“[T]he rapidity with which our Draculas become dated tells us only that every age embraces the vampire it needs” (Auerbach 1995, p. 114). The question then arises as to how the trope was changed to cater to the changing landscape of late communism. By the 1980s, the countries of the Eastern bloc had experienced periods of thaw and now, the state was facing shortages at every level, which contributed to the commodification of the cinema industry. In appropriating Western motifs and themes, communist horror became a “patchwork of styles” (Grabias 2024, p. 130) and a “cross-breeding of […] generic features” (Hanáková 2008, p. 111), leading to “a boon for the revitalization and ideological transformation of the vampire character” (Dolgopolov 2014, p. 46). Under these circumstances, vampire movies served as cautionary tales, warning against the dangers of consumerism and the erosion of traditional gender roles. Falling in the first category are, for instance, Juraj Herz’s 1982 *Upír z Feratu* (*Ferat Vampire*) and Oleg Teptsov’s 1987 *Gospodin oformitel* (*Mister Designer*), which premiered in the USSR, while the second one includes, among others, the Polish *Lubię nietoperze* (*I Like Bats*) (1985), directed by Grzegorz Warchoł and the Russian *Pyushchye krovi* (*The Vampire*), launched by director Yevgeni Tatarsky in 1991.

*Upír z Feratu* and *Gospodin oformitel*—the former released in Czechoslovakia and the latter in the USSR—explore the corruption of technology against the advent of consumerism. In a 1988 article on “The Changing Soviet View of the Future,” Stephen Shenfield notes that since 1980, the Soviet outlook on the world’s social future has become more uncertain and pessimistic, with the previously expected shift of capitalist countries to socialism then seen as improbable (1988, p. 367). This atmosphere of uncertainty bled into the vampire movies of the period, which exhibit an underlying anxiety regarding the future and commodification of technology. A nod to F. W. Murnau’s *Nosferatu* (1922), perhaps the most famous unauthorized adaptation of *Dracula*, the name of the car in *Upír z Feratu* is also a snub at two Western automobile companies, Ferrari and Fiat (Johnston 2021), whose names combine to create the bloodthirsty Ferat, an automobile designed by the sinisterly attractive Madame Ferat and her acolytes to suck the living labor of its race driver consumers. Among its victims is Mima Veberová, an ambulance driver and sports car enthusiast, whose need for speed and friendship with her concerned colleague, Dr. Marek, are exploited by the foreign car maker to advertise the car, first by winning races at the expense of its drivers’ lives and secondly, by secretly filming Dr. Marek protests against the car to garner publicity. However, as Martin Šrajer aptly notes,

it wouldn’t be accurate to see the bloodthirsty car as the film’s main villain. That was the car maker Ferat which used illicit methods to silence their critics and concealed the risks associated with driving their cars. Business was evidently more important than the drivers’ health. After all, […] [the] opening credits invite the viewers to see the film as a laconic criticism of consumerism. […] The sequence combines footage from the world of car racing with the Marlboro logo, an emblem of Western lifestyle. (2024)

However, it is not precisely Western consumerism that the movie denounces, but the communist society’s susceptibility to it. “Herz along with designer Theodor Pištěk chose an existing model from the Škoda Car Museum in Mladá Boleslav” and to promote the feature, “it was displayed in the shop window of the Družba department store at the Wenceslas Square in Prague” for six weeks (Šrajer 2024). The audience knew that the Ferat was not, in fact, a car produced abroad.

Conversely, *Gospodin oformitel* tells the story from the point of view of the creator. Based, like *Upír z Feratu*, on a literary work about an undead vehicle—Herz’s movie is indirectly inspired from Josef Nesvadba’s 1962 short story “Upír ltd” (Vampire Ltd), while Teptsov’s is a loose adaptation of Aleksandr Grin’s 1925 novelette “Seryj Avtomobil” (The Grey Motor Car)—*Gospodin oformitel* shifts the focus from the object of the protagonist’s paranoia, the grey automobile, to the object of his desire, the mannequin Anna/Maria. The vehicle, however, does not disappear from the movie completely; in fact, it appears to be an extension of Anna/Maria, hinting that the socialite—the shop window doll created by Platon Andreevich, the main character—are one and the same entity. Her maker, however, is no Madame Ferat; like Mima, he feeds the mannequin his own blood—and eventually dies in a final interaction with the haunting automobile—to create a shop window doll for a high-end jewelry store. In both *Upír z Feratu* and *Gospodin oformitel*, vampire capitalism dehumanizes creators and consumers alike, leaving behind a hollow existence driven by material aspirations.

“Technology serves to institute new, more effective, and more pleasant forms of social control. […] The totalitarian tendency of these controls seems to assert itself in still another sense – […] by creating similarities in the development of capitalism and communism,” writes Herbert Marcuse in *One-Dimensional Man* (1964, pp. 8–9). However, in the Western movies of the period that explore similar themes—John Carpenter’s 1983 *Christine* and Michael Gottlieb’s 1987 *Mannequin*, both of which were released in the US—vampire capitalism is not a force of evil. Carpenter’s adaptation of Stephen King’s eponymous novel portrays the vehicle commodity “itself [as being] vampiric,” killing a factory worker and an inspector while still on the assembly line (Bacon 2020, p. 126), and in *Mannequin*, Claire Timkin, the owner of the store using Emmy, the doll animated by the spirit of an Ancient Egyptian woman, in the shop display redeems the capitalistic system which fired Jonathan Switcher, the mannequin’s maker, for his lack of productivity, and protects his relationship with the living doll. The final scene, in which Jonathan and Emmy marry in the store window, and the movie’s timely premiere—six days before the National Consumers Week in February 1987—showcase consumer culture as an integral part of American society. Here, the relationship between capitalist, worker, product, and consumer is depicted as giving life rather than sucking it away.

The connection between vampirism and women—in both *Upír z Feratu* and *Mannequin*, the representatives of the capitalist forces are women—is further explored in Grzegorz Warchoł’s *Lubię nietoperze* and Yevgeni Tatarsky’s *Pyushchye krovi*. To understand the trope in this context, it is important to keep in mind that the condition of women in the Eastern bloc differed significantly from one communist country to another. During the mid-1980s, the Catholic Church’s family model came under scrutiny for its strongly traditional views, describing women’s involvement in the workforce as a “necessary evil” that hinders their role as mothers; according to the Church, “the changes in the family taking place in Poland pointed to its erosion and deep crisis” (Siemieńska 1998, p. 129). In this context, *Lubię nietoperze*’s Iza, a happily single vampire-working woman who ultimately pursues mortality and a domestic life, appears as an accurate portrayal of the gender struggle within mid-1980s Polish society. In fact, it might almost come across as a propaganda film were it not for the twist revealing that Iza’s young daughter has inherited her mother’s—and possibly her aunt’s—vampiric gene. Similarly, Warchoł’s portrayal of vampirism holds a double meaning; on the one hand, Iza’s yearning for love seems to suggest that her celibate vampire life is, in fact, a hollow existence, but on the other hand, her vampirism is also the source of her bodily autonomy and empowerment—at night, Iza guises as a sex worker and operates as a vigilante against the sex offenders who target her and other women. This parallel life, while not satisfactory for Iza, is empowering especially when viewed against the period’s real-life social and legal background; in 2020, Agnieszka Kościańska analyzes “Gender on Trial” in Poland from 1970 to the present and opens her study with a 1983 sexual assault perpetrated against a teenager named—coincidentally or not—Iza, where the aggressors were not found guilty because the victim “did not fit neatly into the category of a ‘proper woman:’ “she had had several sexual partners in the past and that she did not feel any guilt while masturbating” and “preferred to spend her time reading […] instead of helping her mother do the cooking and cleaning” (p. 112). In a context shaped by the notion that women are legally and biologically accountable for male sexuality (Kościańska 2020, p. 121), fictional Iza’s vampirism is an instrument for self-preservation.

The premiere of *Pyushchye krovi*, Yevgeny Tatarsky’s movie about a vampiric matriline based on Aleksey Tolstoy’s novella “Upyr” (The Vampire) (1841), took place against a similar background. In “The ‘Women Question’ in the Age of Perestroika” (1991), which appeared, like the cinema adaptation, in the wake of the Soviet Union, Maxine Molyneux notes that Russian feminists highlighted the gendered impact of deteriorating social conditions and rising violence in the USSR, as seen in the increase in domestic abuse and rape (p. 105). Countess Marfa Sergeevna, the matriarch of the vampire family, is the only female character to be depicted unaccompanied (Herbert 2023, p. 73), which suggests that she is at once powerful enough to need no protection and a pariah in relation to the other women in the movie; given her status, she mirrors the condition of Soviet women in high-power positions: in 1990, only 2 to 3% of women held roles in political decision-making bodies (Mespoulet 2006). Like *Lubię nietoperze*, Tatarsky’s movie ends with the vampire being defeated by male intervention, but in a vein similar to Iza, the countess passes down the vampiric gene to her niece, Dasha, whose prospect of living a traditional domestic life with Runevsky, the main character, is also left up for debate.

This ambiguity seems to bleed into the vampire family movies produced in the US, which include Stuart Gordon’s *Daughter of Darkness* (1990), a TV movie filmed in Hungary yet set in Romania right before the collapse of the communist regime. The story follows Katherine, a half human, half vampire American citizen, who travels to Bucharest to find her father only to learn that he is Prince Constantine Cyprian, the sympathetic head of a dying breed of Romanian vampires who championed his country’s openness toward the West in the 1960s.^7^ To save her from Grigore Petrescu, their self-proclaimed leader who wishes to impregnate Katherine to create a new vampiric species immune to daylight, Constantine sacrifices himself, as he did his happiness when he left his daughter and her mother. Helped by the American ambassador to the capital of Romania, she causes a fire in the vampires’ hideout, destroying the last members of the vampire breed. While, in theory, she carries, like Iza’s daughter and Dasha, her father’s bloodthirsty gene, she distinguishes herself from them because the movie ends with her and the ambassador’s falling in love. Katherine, therefore, is suggested to have refused the role of vampire matriarch, fully embracing her human side and a domestic life outside Romania, the oppressive vampire capital of the world.

In the Romanian reviews of the movie, however, neither Prince Constantine’s sacrifice nor Katherine’s refusal of her “higher” vampiric mission has a redeeming quality. Released in 1992, three years after the fall of the communist regime, the movie is criticized for featuring “only two truly human figures, Katherine and the American diplomat,” with the reviewer jokingly sympathizing with their vampire victims when he notes how “[a]s expected, Katherine escapes and, with the help of the American diplomat, of course, manages to wipe out all our vampires by setting them ablaze” (Filip 1992, p. 3).^8^ Another critic seems even more outraged by the movie’s premise; in the final death brought by the two unto the vampire coven, he sees a metaphor for Romanians as a “worthless, violent people—dirty, devoid of any virtues—ripe for assimilation, re-education, and division” (Georgescu 1992, p. 3). Of Prince Constantine, the “Bela Lugosi imitation, albeit [heroic],” who became a target of the secret police after his involvement in the political reform of the 1960s—an aspect duly noted by Jon Burlingame (1990, p. 14)—the Romanian reviewers say nothing of note.

## The vampire in late communist Romanian cinema: oppressor of the working class and foreign threat to national interests

But was Romania really the cradle of vampirism? While Raymond T. McNally and Radu Florescu’s *In Search of Dracula* (1972) was not the first study to associate Romanian voivode Vlad Ţepeş with Count Dracula, Bram Stoker’s fictitious bloodsucker (Miller 2011, p. 211), but the lack of competing expertise allowed them to shape the discourse and leave their mark on the topic (Leatherdale 2011, p. 4). The background of the release may have also contributed to the volume’s success, which amounted to over 200,000 copies in 1975:^9^ no less than 32 cinema productions recorded by the Internet Movie Database between 1960 and 1971 are associated with the keyword “Dracula,” and until half a year before its publication, Romania enjoyed an unprecedented level of openness toward the West. The success of *In Search of Dracula* quickly led to the creation of a joint American-Romanian tourism initiative—an 18-day guided tour called *Spotlight on Dracula: An Adventure in Transylvania*, which focused on sites linked to Vlad the Impaler and northeastern Transylvania, the setting of Stoker’s novel (Light 2012, p. 65). However, while actively monetizing the link between Vlad Ţepeş and Dracula, “Romanian tourist offices abroad began to print tourist leaflets emphasizing the historical figure of Vlad Ţepeş” and “[…] Constantin C. Giurescu, a famous historian, gave a lecture on Vlad Ţepeş during a conference tour at US universities” to counteract what local authorities considered to be a defamation campaign against one of the country’s historical figures (Gheonea 2022, p. 108). Their efforts, however, did not bear fruit; despite the involvement of the Romanian Secret Police in the “Dracula Question,” the year 1975 saw the release of a documentary based on *In Search of Dracula*, a documentary “in which Christopher Lee took the part of Vlad Tepes. The use of an actor who was at that time almost inseparable from the role of Count Dracula cemented the link” (Miller 2011, p. 212). In 1979, when no less than five films associated the Romanian voivode with Stoker’s antagonist were released abroad, the Romanian Ministry of National Defense collaborated to release *Vlad Ţepeş*, a historical drama which had been planned as early as 1974 (Gheonea 2022, p. 110).

“In an August 1977 interview with the [Romanian magazine *Cinema*], the movie’s screenwriter reveals their intent to challenge the Western portrayal of Vlad Ţepeş, which had been skewed by sensationalism and commercial interests” (Jitea 2023). As Bogdan Jitea explains, *Vlad Ţepeş* depicts the Romanian ruler as the visionary behind a campaign to establish a Golden Age in Romania—later embraced by communist leader Nicolae Ceauşescu five centuries later—while attributing accounts of his cruelty to fabrications by “Transylvanian Saxon merchants, who, seeking revenge for the violation of their commercial privileges, created and disseminated this villainous image within German circles” (2023). Intended to rehabilitate Vlad Ţepeş’s reputation, this movie was, perhaps, also meant to shed a more positive light with Ceauşescu’s own association with Vlad Ţepeş and, by extension, the vampire Dracula; Valentin Gheonea notes that “[b]efore the official premiere, the film was watched by Nicolae Ceausescu, who granted his consent for the release (2022, p. 110). Like earlier attempt to divorce the villain from the historical hero, this effort failed; during the 1989 Revolution, which saw Ceauşescu’s violent descent from power, “[i]n Bucharest and in other cities the demonstrators shouted, ‘Down with the vampire!’’’ (Codrescu 1991, p. 71)

Similarly based on a source material linking Romania with vampires was also Vlad Bitca’s 1975 TV play *Castelul din Carpaţi* (The Castle of the Carpathians). A loose adaptation of Jules Verne’s 1892 *Le Château des Carpathes* (*The Castle of the Carpathians*), which was translated no less than nine times in total into Romanian—but only once during the communist regime—*Castelul din Carpaţi* reimagines the story of an energy vampire baron who terrorizes a Transylvanian village and immortalizes an Italian *prima donna*’s voice with his servant’s inventions into a depiction of class struggle; specifically, the ending depicts his unpaid aide, Orfanello, destroying his master’s machine, which keeps the opera singer alive, and saving the castle intruder-turned-prisoner, Radu Gorj, a rational Wallachian geologist: the worker and the intellectual unite to take down the exploitative aristocrat.

In a movie adaptation with the same title from 1981, which eliminates the supernatural element altogether, the plot is further appropriated politically to emphasize the plight of Romanians in Hungarian-ruled Transylvania, then involved in a highly visible emancipation movement which Verne alluded to in his novel (Martin 2023, pp. 73–88). Yet although its director, Stere Gulea, is now associated with anti-communist pictures such as the 2013 *Sunt o babă comunistă* (*I Am an Old Communist Hag*) and the 2018 *Moromeţii 2* (*Moromete Family: On the Edge of Time*), his adaptation of Verne’s work focuses instead on the historical event mentioned earlier and introduces a new character, an Austro-Hungarian secret agent, who exploits the count’s assistant’s desire for fame and money, to set his employer, a former 1848 revolutionary, and the main character, who fights for the rights of Transylvanian Romanians, against each other and sabotage the latter’s efforts of publishing a petition advocating his cause. In a context where Ceauşescu’s Romania was becoming increasingly isolated and with Romanian Secret Police general Ion Mihai Pacepa having defected to the US a few years earlier, the vampiric secret agent Friecke comes across as a reflection of Romania’s distrust of outsiders and foreign intervention in the local governance.

In contrast, as shown in the analysis of Gordon’s *Daughter of Darkness*, the wake of communism in Romania gave momentum to Western directors, who repurposed the turbulent last days of the regime to explore Dracula’s myth in a contemporary Gothic key.^10^ The first vampire movie to be lensed in post-communist Romania, Ted Nicolaou’s *Subspecies* (1991) tells the story of two American college graduates who visit a former colleague from Romania, where they have a fateful encounter with Stefan Belescu, the half vampire, half human son of Vlad Dracula and his brother, Radu, a Nosferatu-like bloodsucker.^11^ Like Gordon before him, Nicolaou envisions a struggle between “good” and “evil” vampires, with the amendment that, in this case, the vampire king is portrayed as a heroic figure, who defended his human subjects against the Turks, with the former eventually providing him with a bloodstone to satiate his thirst and organizing festivals in his honor. While offering an exoticized perspective of early 1990s Romania—it is still a backward country, with the American students hoping that their lodging has a bathroom—it should be noted that *Subspecies* casts a positive light on Stefan’s “human(e)” vampirism. A zoologist educated abroad, he is, like Prince Constantine in *Daughter of Darkness*, a civilizing agent, who, while unable to change his nature, demonstrates that his links with the West help keep his condition in check.

Once again, however, this message fails to be acknowledged in the Romanian press, even though Nicolaou worked with a company from Bucharest to produce it. If the Romanian critics recognized the “final utility” of features like *Subspecies* (Căliman 1992, p. 23), moviegoers deemed the work as “offensive” for imagining “a subspecies group known as Romanians decapitating their dead and driving a stake through their hearts to prevent them from becoming vampires” (Doaga 1992, p. 4). *Subspecies* was similarly received in the US, as well; while one reviewer commends it for “trying to get back to the folklore roots of vampirism” (Scheib 1996), the American audience seems to have been struck by the same elements as their Romanian counterpart. In the words of one viewer quoted in a Romanian daily, “it seems you [Romanians] are a very backward people. Romania is the birthplace of Dracula, one of the bloodiest vampires to ever exist. […] [I]n a place called Transylvania, […] the locals decapitate corpses when they suspect vampires might be hiding in their bodies” (Neacşu 1992, p. 8).

## Conclusions

In analyzing the different iterations of the vampire trope in communist cinema, this article shows that the understanding of the myth was not monolithic in the Eastern bloc. In fact, Marx’s interpretation of the trope as alluding to capitalism and capitalists bled into socialist vampire movies only very early and very late in the history of communist cinema, when Eastern bloc directors forged new narratives in line with state ideology and when socialist countries were grappling with reconciling their lifestyle with the ever more present Western influences. Outside of these temporal poles, the vampire iterations of communist cinema took inspiration from folklore, appropriating creatures from oral culture or rural literature to criticize the most terrifying eras in Soviet history.

This paradigm changed in late communism, not only in the countries of the Eastern bloc, which experienced the final days of their socialist regimes, but also in the West, where vampire movies, some of them shot in communist states, envisioned stories about struggles between “good” and “evil” vampires, where the former, although inspired by Stoker’s Transylvanian Count, are portrayed as overcoming their condition through exposure to the ways of the West. An exception to the vampire narratives of late socialist cinema, Romanian movies failed to embrace the trope and actively opposed it, even if the state authorities sought to capitalize on the association between Dracula and Vlad the Impaler. In the few instances they adapted vampire literature for the screen, this supernatural element was, in most cases, heavily abridged, or treated, at best, as an aside to other themes of their source texts, which were brought to the forefront to emphasize the country’s heroic history and its struggles for independence from foreign powers.

The reception of *Daughter of Darkness* and *Subspecies* in both the US and Romania highlights the crucial role audience perception plays in shaping a movie’s impact and actual interpretation. While Western directors used the vampire metaphor to explore Romania’s post-communist transition within a Gothic framework—albeit optimistically, as both movies eventually propose a solution to the vampiric condition, a metaphor of the country’s oppressive past—Romanian audiences and critics largely overlooked the denouements. The two movies’ primary audience—American viewers—likely grasped the metaphors of East-West tensions and ideological struggle, particularly within the geopolitical context of the early 1990s. However, the Romanian reception underscores how cultural context shapes interpretation. What may have seemed like an obvious allegory to US audiences—vampirism as the embodiment of oppressive regimes or cultural stagnation—was perceived differently in Romania, where it was seen as an unfair or even hostile portrayal of the country’s struggles and history alike.

## Data Availability

Data sharing is not applicable to this article as no datasets were generated or analyzed during the current study.

## Footnotes

1 The Marxist iteration of the vampire appears to have been the most popular apprehension of the word in at least one of the communist states discussed in this article. “[T]he most famous iteration of the term in Romanian literature, the humorous blunder on “vampire” in [Ion Luca] Caragiale’s 1885 comedy *O scrisoare pierdută* [The lost letter], […] draws on the metaphor of the vampire-exploiter. […] Caragiale might have learned of this metaphor from his friend, Romanian Marxist thinker Constantin Dobrogeanu-Gherea” (Martin 2024, p. 3).

2 All translations are my own unless otherwise noted.

3 It loosely adapts Prosper Mérimée’s 1869 novella *Lokis*.

4 “With its folkloric imagery, Stalinist cinema domesticated national difference, while maintaining the spectacle of particular spaces and the peoples that inhabited them” (First 2015, p. 29). Relevant examples in this regard include Sergei Parajanov’s *Andriesh* (1954), shot in Kiyv by a Soviet Georgian director and starring a Moldovan actor in the title role. However, the ethno-national themes explored in the cinema of the second half of the 1960s lost this political agenda, and “the only ideological component […] here [in this genre] is the confrontation between Stalinism and the Thaw” (Kim 2022, p. 15).

5 See Count Mitterhaus in Hammer Productions’ *Vampire Circus* (1972) and Count Yorga in *Count Yorga, Vampire* (1970), released in the UK and US, respectively.

6 The protagonist of the Italian *Il cav. Costante Nicosia demoniaco, ovvero: Dracula in Brianza* (*Dracula in the Provinces*) (1975) and Count Dragalescu’s victim is the snobbish Italian heir Constante Nicosia, while Count Erik Dracultre preys on female Spanish entertainers in *Las Alegres Vampiras de Vögel* (*Vampires of Vogel*), which premiered in Spain in the same year.

7 Indeed, like other communist states, Romania aimed to show a willingness to engage with the West in the 1960s. “Romania was the first country in the Soviet bloc to host the President of the United States when Richard Nixon visited Bucharest in 1969. Another clear manifestation of Romanian independence in the international arena was Ceauşescu’s condemnation of the invasion of Warsaw Pact troops into Czechoslovakia in 1968” (Anonymous).

8 All translations are my own unless otherwise noted.

9 This figure is quoted from the front cover of Raymond T. McNally’s *A clutch of vampires*, published by Warner Books in 1975.

10 Pop culture products with vampires accompany time again turning points in Romanian history. An illustrative example in this regard is G.M. Amza and Alexandru Bilciurescu’s *Vampirul* (The Vampire), which was published in 1938, when Romanian society, still young at the time, was grappling with the rapid developments of the interwar period. *Vampirul* “is not only the first Romanian novel to feature a vampire antagonist explicitly but also a work whereby the co-authors do not propose a local Gothic formula but a Gothic crime fiction product, which revisits the ‘foreign plot’ of *Dracula* and reinterprets it in accordance with the era’s vampire cinema tropes. However, unlike *Dracula* and its 1930s cinematic iterations, whose ‘foreign plots’ envision the vampire either as an undead aristocrat or as a pretend monster, in *Vampirul* the two dimensions of the trope combine to produce a ‘local vampire’ who evokes East-Central European representations of the monster as heretic members of the clergy” (Martin and Baghiu 2024, p. 7).

11 Nicolaou loosely adapts Vlad Ţepeş’s biography by referencing the Romanian voivode’s father, Vlad II Dracul (1436–1442) and half-brother, Radu cel Frumos (c. 1438–1475), with whom he had a troubled relationship, an aspect also depicted in *Subspecies*.
